# Conditions for Waveblock Due to Anisotropy in a Model of Human Ventricular Tissue

**DOI:** 10.1371/journal.pone.0141832

**Published:** 2015-11-02

**Authors:** Nina N. Kudryashova, Ivan V. Kazbanov, Alexander V. Panfilov, Konstantin I. Agladze

**Affiliations:** 1 Life Science Center, Moscow Institute of Physics and Technology, Dolgoprudny, Russia; 2 Department of Physics and Astronomy, Ghent University, Ghent, Belgium; Georgia State University, UNITED STATES

## Abstract

Waveblock formation is the main cause of reentry. We have performed a comprehensive numerical modeling study of block formation due to anisotropy in Ten Tusscher and Panfilov (2006) ionic model for human ventricular tissue. We have examined the border between different areas of myocardial fiber alignment and have shown that blockage can occur for a wave traveling from a transverse fiber area to a longitudinal one. Such blockage occurs for reasonable values of the anisotropy ratio (AR): from 2.4 to 6.2 with respect to propagation velocities. This critical AR decreases by the suppression of *I*_*Na*_ and *I*_*Ca*_, slightly decreases by the suppression of *I*_*Kr*_ and *I*_*Ks*_, and substantially increases by the suppression of *I*_*K*1_. Hyperkalemia affects the block formation in a complex, biphasic way. We provide examples of reentry formation due to the studied effects and have concluded that the suppression of *I*_*K*1_ should be the most effective way to prevent waveblock at the areas of abrupt change in anisotropy.

## Introduction

Cardiac arrhythmias are one of the largest causes of death in the industrialized world [1]. In most cases, lethal cardiac arrhythmias arise from abnormal wave propagation and are driven by reentrant sources of excitation, or vortices [1]. Such vortices occur as a result of blocks of wave propagation [2–4]. Thus, understanding the conditions of block formation is a central issue in the theory of cardiac arrhythmias.

There are several mechanisms of waveblock formation. Historically, the first proposed mechanism was the formation of a block due to additional tissue stimulation at the tail of the propagating wave [5], the so-called *S1S2* stimulation. Later, two main classes of unaffected break formation mechanisms were identified. The first class deals with the wavebreaks that occur at the heterogeneity of cardiac tissue, which can either be due to the different properties of cardiac cells in different regions [6, 7] or to dynamical instabilities [8–10]. The second class deals with geometrical heterogeneities, in which wavebreaks can either occur as a result of the detachment of a wave from a boundary with a sharp end [11–13] or can be formed around obstacles [14, 15]. Another widely studied geometrical structure is called a “gate”, or isthmus, where waveblocks can be formed at the locations of abrupt tissue expansion [16, 17].

In all cases involving geometrical heterogeneity, the wavebreak occurs as a result of a so-called “current-to-load mismatch”. The wave propagation is blocked when the density of the current produced by a wavefront in a given region is not sufficient to initiate excitation in the adjacent tissue. In the cases listed above, the current-to-load mismatch occurs either due to the curvature of the wavefront or as a result of the abrupt extension of the domain ahead of the wavefront. In all of these cases, the length of the wavefront abruptly increases, resulting in a decrease in the current density and propagation block.

There is also another possibility for the current-to-load mismatch: it may occur if the local resistivity of cardiac tissue changes. Indeed, if a wave propagates from a region with high resistivity to a region with low resistivity, the same current will produce a lower voltage gradient, making wave propagation through the boundary more difficult. Such a change in resistivity naturally occurs due to tissue anisotropy and can potentially result in a propagation block. This was demonstrated in our previous study [18] in experiments with a cell culture of neonatal rat myocytes, and numerically in a low dimensional model for cardiac tissue. Here, we extended the theoretical study to a detailed ionic model of human cardiac cells. We found the conditions for the waveblock formation, studied how the conditions depended on the conductivity of the main ionic currents in the cardiac tissue, and provided examples of the formation of ectopic beats and reentry due to abrupt changes in anisotropy. We discussed the possible importance of this effect to the onset of cardiac arrhythmias.

## Materials and Methods

### 0.1 Electrophisiological model

We used ten Tusscher and Panfilov cell model (TP06) for human ventricular cardiomyocytes [19, 20]. In this model, cardiac electrophysiology is described with the following monodomain equation:

$$\begin{array}{c} \frac{\partial V}{\partial t}=\nabla (\sigma (\vec{x})\nabla V)-\frac{I_{ion}\left(V,\ldots \right)}{C_{m}}, \end{array}$$ (1)

$$\begin{array}{c} \sigma \left(x<x_{c}\right)=(\begin{array}{cc} \sigma_{⊥} & 0\\ 0 & \sigma_{∥} \end{array});\;\sigma \left(x>x_{c}\right)=(\begin{array}{cc} \sigma_{∥} & 0\\ 0 & \sigma_{⊥} \end{array}); \end{array}$$ (2)

where *V* is a transmembrane potential, *σ* is a tensor of coupling coefficients, *x*_*c*_ is a coordinate of the border, and *I*_*ion*_ is a sum of ionic currents given by the following equation:

$$\begin{array}{c} I_{ion}=I_{Na}+I_{K1}+I_{to}+I_{Kr}+I_{Ks}+I_{CaL}+I_{NaCa}+I_{NaK}+\\ +I_{pCa}+I_{pK}+I_{bCa}+I_{bNa}. \end{array}$$ (3)

For most of the parameters of the TP06 model, which describes normal conditions, we used values listed in Tables 1 and 2 of [20], which correspond to epicardial cells. We also decreased the conductivity of the ionic channels to 75%, 50%, and 25% percent of their normal value to study the effect of the channel blockers on the unidirectional block formation. To model hyperkalemia, we varied [*K* ^+^]_*o*_ from 5.4 mM to 20 mM.

### 0.2 Numerical methods

To solve the differential Eq (1) in 2D (and 1D) we used an explicit Euler scheme:

$$\begin{array}{c} \frac{V_{i,j}^{k+1}-V_{i,j}^{k}}{\tau }=\frac{1}{h^{2}}(J_{i+\frac{1}{2},j}-J_{i-\frac{1}{2},j}+J_{i,j+\frac{1}{2}}-J_{i,j-\frac{1}{2}})-\frac{I_{ion}\left(V_{i,j}^{k},\ldots \right)}{C_{m}} \end{array}$$

where the time step *τ* = 0.005 *ms*, the space step *h* = 125 *μm*, and flow $J_{i+di,j+dj}=\sigma_{i+di,j+dj}\left(V_{i+2di,j+2dj}^{k}-V_{i,j}^{k}\right)$, where *di*, *dj* ∈ (−1/2, 0, 1/2) and one of them is equal to zero. For example for *J*_*i*+1/2,0_, *di* = 1/2 and *dj* = 0, etc. Here superscript indexes are related to time, and subscript—to space. Transmembrane potential was determined at the mesh points, whereas flow and coupling coefficients were set in half-integer nodes. In 2D simulations, we used (*τ* = 0.02 *ms*, *h* = 250 *μm*), which is within the widely accepted values for ionic models [21]. We also checked key results of our paper in simulations with (*τ* = 0.0008 *ms*, *h* = 50 *μm*) and showed that the results of our study on waveblock formation also hold for this spatial resolution.

We applied Neumann (’no flux’, *J* = 0) boundary conditions in all simulations.

The model was implemented using C and CUDA programming languages. Results were obtained with an NVIDIA Tesla K20 GPU.

### 0.3 Computational setup

**Fig 1:**
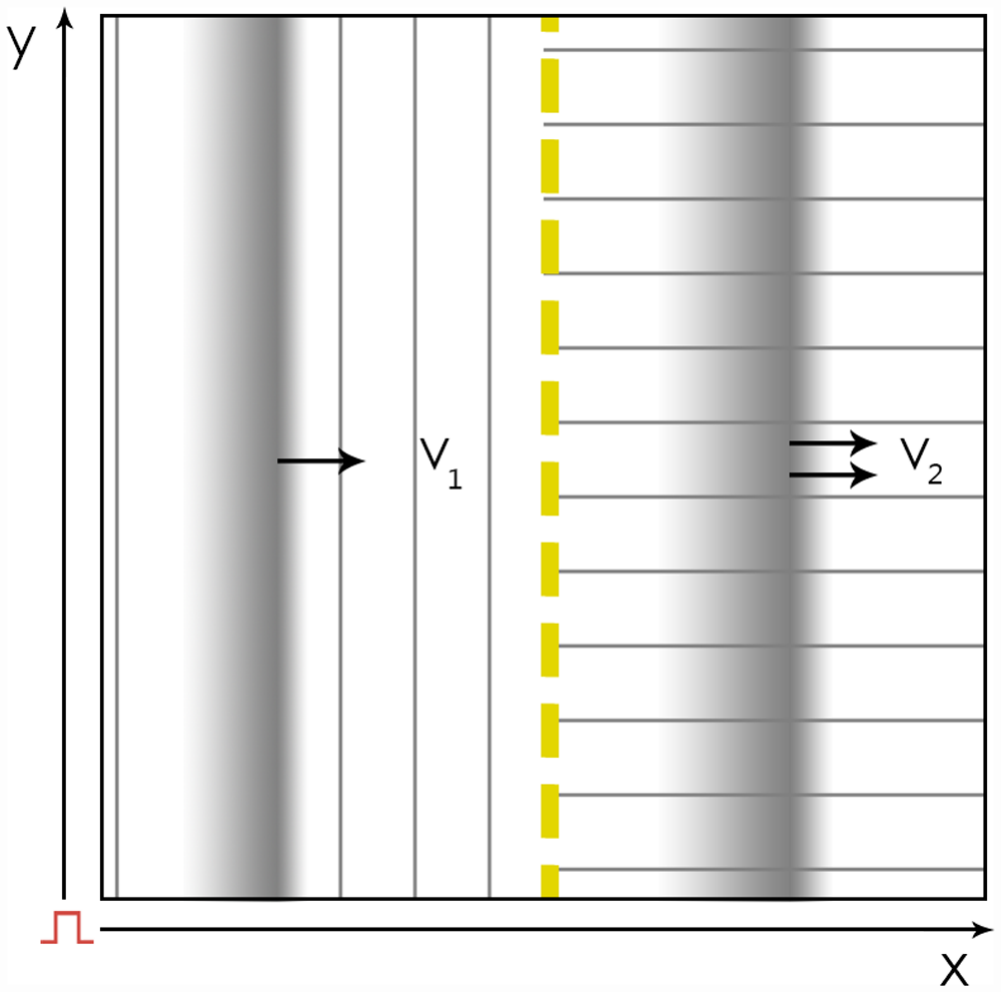
Schematic representation of the computational setup in 2D. The tissue is divided into two parts with an orthogonal fiber orientation. The boundary is shown by the yellow dashed line. In the left part, the fibers (represented by thin lines) are parallel to the boundary, and in the right part, the fibers are orthogonal to the boundary. Two propagating waves are schematically shown as greyscale images. When stimulation was applied to the left border, wave propagation was observed to be translationally symmetric along the y axis and could be studied in 1D simulations.

We studied the extreme case of a border between two areas with orthogonal fiber alignment, shown in Fig 1. Fibers were parallel to the border in one part and perpendicular in another. The anisotropy ratio (AR) was assumed to be the same in the whole sample. Thus, the orthogonal fiber orientation provides the greatest jump in velocity on the border, so the effects caused by this border would be the most pronounced.

First, we studied plane wave propagation across the border. In this case, the wave front is parallel to the border, and the whole system can be reduced to a 1D case because of the translational symmetry along the vertical y-axis (the solution does not depend on y).

Second, we returned to the 2D representation to study non-symmetrical wave propagation, such as point stimulation and reentry formation.

In both the 1D and 2D simulations, the equations were first integrated without stimulation for 50 seconds in order to obtain spatially uniform steady state values, which were saved and used as the initial conditions for all further simulations. In studies of periodic wave propagation, we ignored the waves obtained by the first three stimulations to minimize the possible side effects of transient processes. Stimulation was applied in a rectangle 6 px wide along the left border of the sample. In this rectangle, transmembrane potential was immediately set to 50 mV.

The key parameter that characterizes the probability of waveblock formation is the AR, the ratio of the velocities parallel and perpendicular to the fibers. It could be derived from the coupling coefficients as follows:

$$\begin{array}{c} AR=\frac{v_{∥}}{v_{⊥}}=\sqrt{\frac{\sigma_{∥}}{\sigma_{⊥}}}. \end{array}$$ (4)

In simulations, where the AR was varied, *σ*_∥_ was fixed and equal to 0.154 *mm* ^2^/*ms* [19], and *σ*_⊥_ was set to *σ*_∥_/(*AR* ⋅ *AR*).

## Results

**Fig 2:**
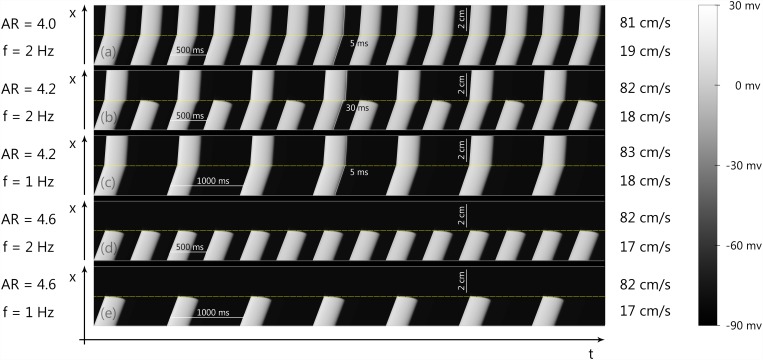
Wave propagation through the boundary of abrupt change of anisotropy in cardiac tissue for various ARs and frequencies of stimulation (f). Figure shows space(x)—time(t) plots of wave propagation along 1D cables with abrupt changes of conductivity, representing abrupt changes of fiber orientation in 2D cardiac tissue. The details of the computational setup are represented in Fig 1 and in section 0.3. (a, c) no block occurs, (b) 1:2 block, (d, e) complete block at the boundary.

Fig 2 shows a typical process of wavebreak formation. We see that in the case of relatively small anisotropy (AR = 4.0, see Fig 2a), the wave passes through the boundary of heterogeneity with only a small delay at the border (approximately 5 ms). However, if the AR is increased to AR = 4.2 (Fig 2b), every second wave is blocked at the border, and those waves that pass are delayed for 30 ms. The decrease in stimulation frequency eliminates the block (Fig 2c). If, however, we increase the AR to AR = 4.6, each wave will be blocked at the boundary regardless of the frequency of stimulation (Fig 2d and 2e).

Overall, we found that we can always obtain a waveblock at some critical AR, and its value decreases with an increase in the frequency of stimulation. In addition to frequency, other factors can also affect block formation. In the next section, we examine how the conductivity of main ionic channels and external conditions, such as elevation of the outer potassium concentration ([*K* ^+^]_*o*_), typical for ischemia, affect the formation of blocks at the anisotropic boundary.

### 0.4 Channel blockers

**Fig 3:**
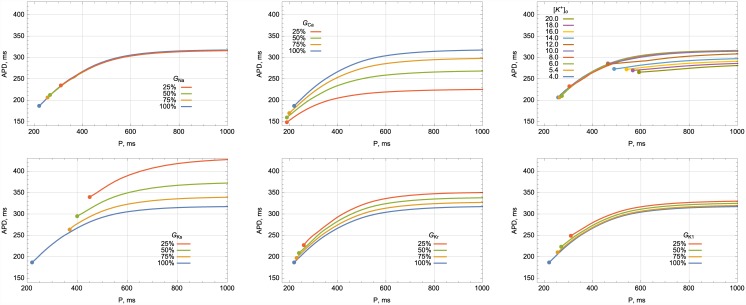
*APD*_90_ restitution curves for various conductivities of ionic currents and external [*K* ^+^]_*o*_ concentration. (a) Inhibition of *I*_*Na*_; (b) Inhibition of *I*_*Ca*_; (c) Hyperkalemia. (d) Inhibition of *I*_*Ks*_; (e) Inhibition of *I*_*Kr*_; (f) Inhibition of *I*_*K*1_; The graphs show the dependency of *APD*_90_ on the period of stimulation. Data from S1 Table was used. The blue line on each graph corresponds to the normal parameter values. Different colors show the curves for decreased values of the various ionic conductivities or external [*K* ^+^]_*o*_ concentration, marked in the frame of each sub-figure. Frequencies higher than 2.7 Hz were obtained by gradually decreasing the period by 5 ms per cycle.

Here, as in the previous simulations, we considered two areas with orthogonal fiber orientation and stimulated a plane front propagation through the boundary from the transverse to the longitudinal fiber orientation. The wave front was parallel to the border between these areas. We decreased the conductivity of various ionic currents to 75%, 50%, and 25% of their normal values and calculated the dependence of the basic cycle length (BCL) on the critical AR (see S2 Table). In Fig 3, we present the data on how such changes in the conductivity of ionic currents and other parameters used in our simulation affect the duration of an action potential.

#### Sodium current *I*_*Na*_

**Fig 4:**
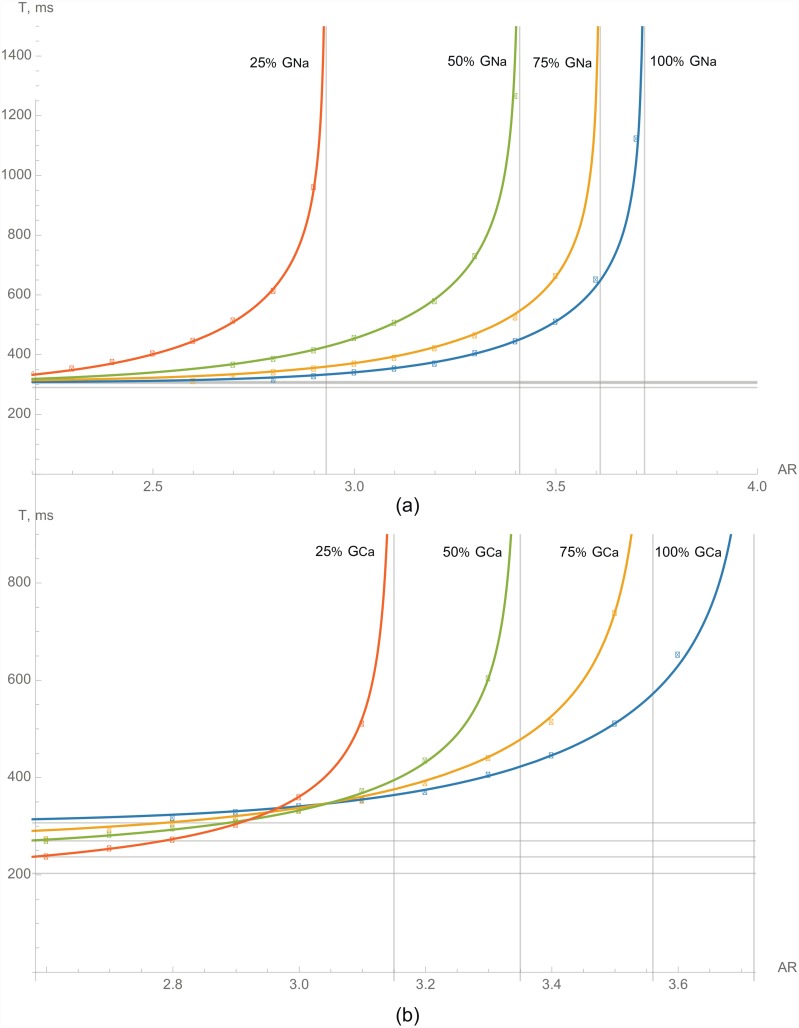
The dependencies of the critical period of stimulation on the AR for normal cardiomyocytes (blue line), with the addition of various channel blockers. Inhibition of (a)—*I*_*Na*_, (b)—*I*_*Ca*_.

Fig 4a demonstrates how frequency and *I*_*Na*_ affect the critical AR. We see that at low frequency and normal *I*_*Na*_ (the blue line), the critical ratio is 3.7. However, if the frequency increases to 2.5 Hz, the ratio decreases to 3.3. The minimal stimulation period under normal conditions in TP06 is about 300 ms, and for high frequencies, the critical AR decreases dramatically to approximately 2.9 and becomes heavily dependent on the stimulation rate. We also see that the decrease in *G*_*Na*_ to 75%, 50%, and 25% progressively decreases the critical AR. This occurs for all frequencies of stimulation. For short periods, we also see that a block of *I*_*Na*_ results in a decrease of the critical AR, and the curves for lower values of *I*_*Na*_ are slightly above the curves for higher *I*_*Na*_ values. Quantitatively, for 75% of *I*_*Na*_, the critical AR changes to 3.6 for long periods (3% decrease) and to approximately 2.7 for short periods (7%). If the *G*_*Na*_ is further decreased to 50%, the ratio progressively decreases to 3.4 (9%) and 2.4 (17%) for longer and shorter periods respectively, and for 25% of *G*_*Na*_, the typical change is around 26% to 32%.

Therefore, one can see that as both frequency and *G*_*Na*_ reduce the critical AR, the probability of block formation consequently increases. All of these results can be easily explained: the decrease in sodium current *I*_*Na*_ causes a decrease in the excitability of cardiac cells and results in a block formation.

#### Calcium current *I*_*CaL*_

Next, we studied how a decrease in *I*_*CaL*_ affects block formation (Fig 4b). Similar to the block of *I*_*Na*_, we saw changes in vertical asymptote positioning that corresponded to a decrease of the critical AR for a long stimulation period. However, for short periods, the situation was different. We found that in a short period of stimulation, the critical ratio decreased with an increased block of *I*_*CaL*_, and the curves shown in Fig 4b intersected each other around a certain point (3.0, 340).

We can explain such behavior as follows: the shifting of a vertical asymptote is caused by the effect of *I*_*CaL*_ on the wavefront, and the decrease in *I*_*CaL*_ reduces the excitability of the tissue, thus inducing the block at a smaller AR. An additional effect of the block of *I*_*CaL*_ is a shortening of APD (Fig 3). For long stimulation periods, this effect is not essential, as there is sufficient time for the cells to recover their properties before the next wave arrives. However, in short periods of stimulation, this shortening becomes essential, and as a result, the tissue with lower *I*_*CaL*_ for the same short period of stimulation is better recovered and its wavefront is thus more stable. Therefore, one can see that at high frequencies (short periods), the critical AR increases with a block of *I*_*CaL*_.

#### Potassium delayed rectifier currents *I*_*Kr*_, *I*_*Ks*_

**Fig 5:**
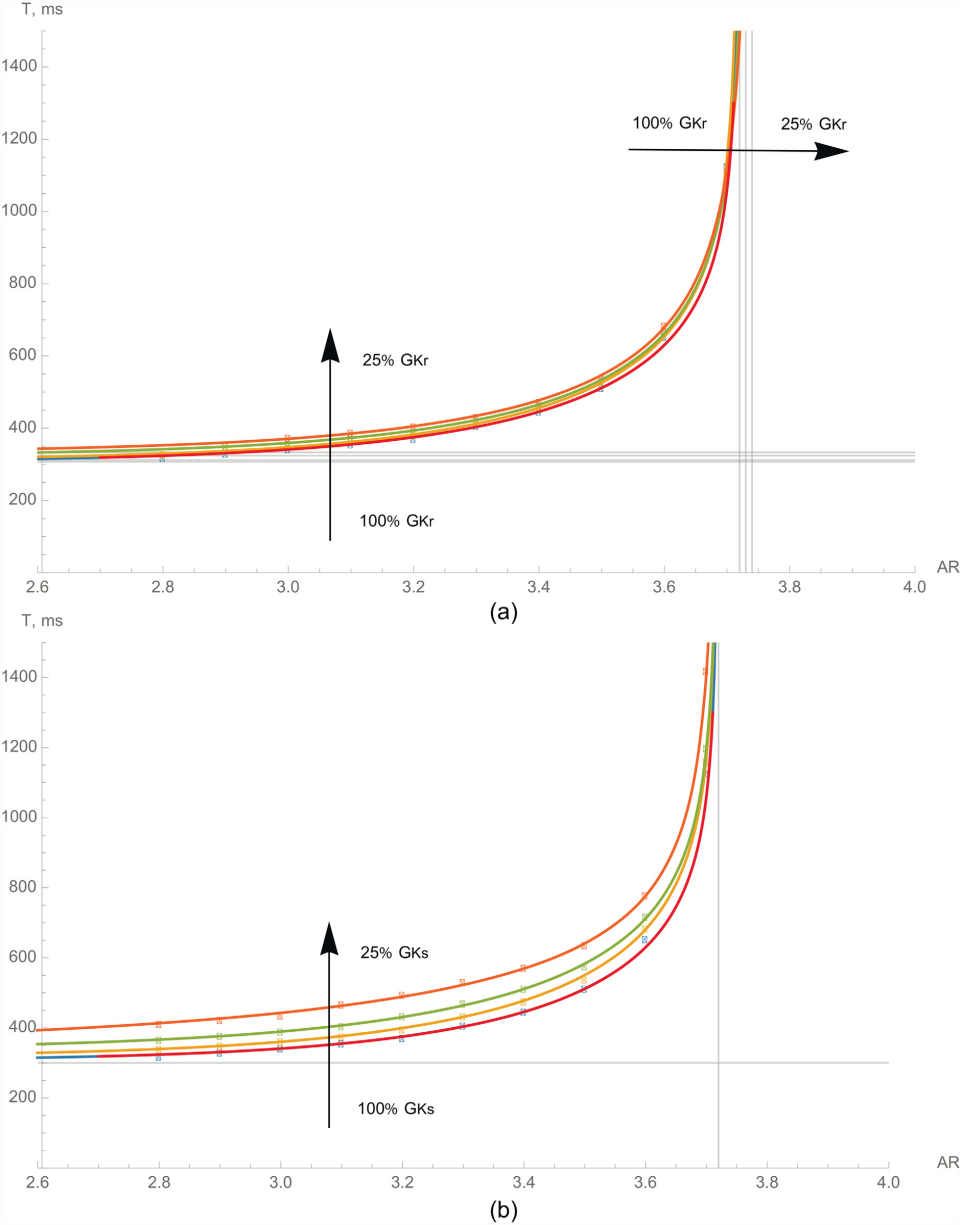
The dependencies of the critical period of stimulation on the AR for normal cardiomyocytes (red line) and with various potassium currents suppressed. Inhibition of (a)—*I*_*Kr*_ and (b)—*I*_*Ks*_. The vertical asymptotes to these plots correspond to the critical AR for a single travelling pulse.

We have also studied how the inhibition of various potassium currents affects block formation (Fig 5). We have decreased *I*_*Kr*_ and *I*_*Ks*_ separately to 75%, 50%, and 25% of their normal values. Rapid delayed rectifier current *I*_*Kr*_ (Fig 5a) has almost no impact on block formation. The critical AR changes slightly from 3.72 at normal conditions to 3.74 when only 25% of *G*_*Kr*_ is available (0.5% change), and APD slightly elongates (*APD*_90_ at 1 Hz becomes 8% longer; see Fig 3). Slow delayed rectifier current *I*_*Ks*_ (Fig 5b) does not change the asymptote position, which means that in a long period of stimulation, such a current does not affect block formation. However, at high frequencies (short periods), the critical AR decreases slightly. Similarly to what we saw in a block of *I*_*CaL*_: *I*_*Ks*_, inhibition elongates APD (Fig 3), and thus, at high frequency, a waveblock occurs for a lower AR.

Hence, both *I*_*Kr*_ and *I*_*Ks*_ do not influence the upstroke directly and have almost no effect on block formation at low frequencies, whereas at high frequencies, all of the changes are based on interaction with the refractory tail, which is mainly determined by the effect of these channels on the APD (Fig 3).

#### Inward rectifier current *I*_*K*1_

**Fig 6:**
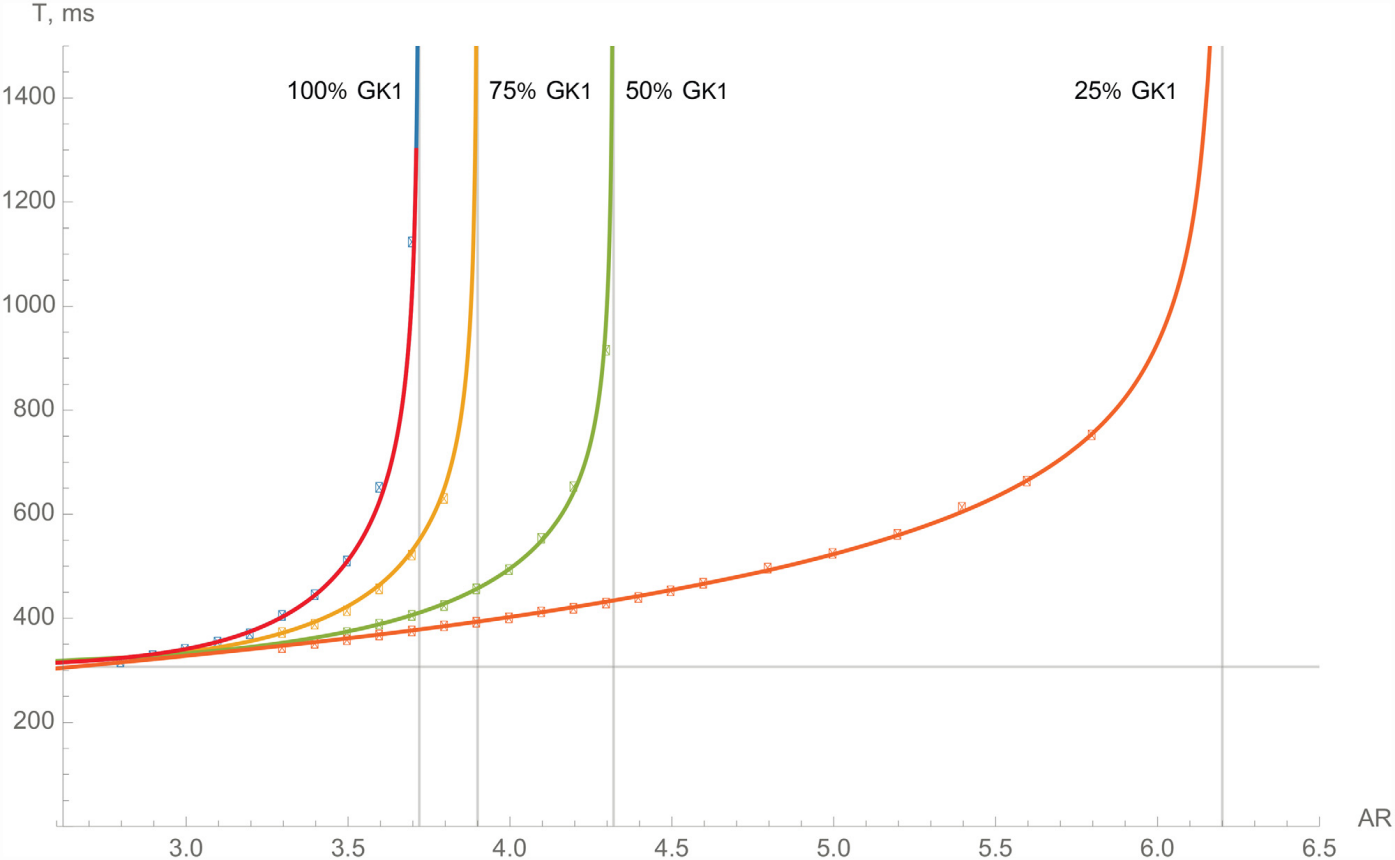
The dependencies of the critical period of stimulation on the AR for normal cardiomyocytes (red line) and with the inward rectifier potassium current *I*_*K*1_ suppressed.

With the decrease of *I*_*K*1_ (Fig 6), we see a significant change in the asymptote’s position, shifting from 3.72 for normal conditions to 3.90, 4.32, and 6.20 for 75%, 50%, and 25% of *G*_*K*1_ respectively. Therefore, we can conclude that a reduction in the conductivity of *I*_*K*1_ substantially reduces the wave block’s ability to form. Indeed, for an anisotropy of 3.7, the block occurs at 0.9 Hz stimulation under normal conditions, whereas for reduced *I*_*K*1_, it occurs at 1.9 Hz for 75%, 2.5 Hz for 50%, and 2.7 Hz for 25% of *G*_*K*1_.

The major difference between the inward rectifier current *I*_*K*1_ and other potassium currents *I*_*Kr*_/*I*_*Ks*_ is that *I*_*K*1_ not only influences the repolarization, but also has a significant effect on the upstroke of the action potential. Moreover, *I*_*K*1_ does not have time-dependant gate variables and works as an outward current between −70 mV and 0 mV with a peak at −50.86 mV [22]. The TP06 model accurately mimics the current-voltage characteristics of this current [19]. Consequently, *I*_*K*1_ affects the APD via two mechanisms, first during the wavefront, and second in the repolarization phase, restoring action potential.

The decrease of *I*_*K*1_ on the one hand, therefore, raises the excitability and eliminates the block due to its decreased impact on the wavefront. On the other hand, it depolarizes the cell, resulting in a lower availability of sodium channels and provoking a block. In our simulations, the first effect was more pronounced, and if *G*_*K*1_ was between 15% and 100% of its normal value, the AR rose with the decrease of *G*_*K*1_. If *G*_*K*1_ was further decreased (below 10%), the cells were depolarized and demonstrated self-oscillations. In this research, however, we limited our study to moderate changes of the ionic current, and we concluded that a moderate inhibition of *I*_*K*1_ to 15–50% prevents block formation at low frequencies of stimulation and does not notably affect block formation at high frequencies of stimulation.

In order to investigate why the inhibition of *I*_*K*1_ changes the critical anisotropy in an unexpected way, we performed additional simulations that highlighted the effects of *I*_*K*1_. In particular, we switched off *I*_*K*1_ at different phases of action potential formation and observed whether such modification eliminated the effects of *I*_*K*1_ change. As we can see in Fig 6, the critical *AR* ≈ 6.0 at 1Hz and 25% *G*_*K*1_, while at 100% *G*_*K*1_ and 1Hz, the block occurs at *AR* ≈ 3.7.

First, we blocked *I*_*K*1_ only at the wavefront (set its conductance to 25% *G*_*K*1_ for *dV*/*dt* > 0), while for all other phases, we used 100% *G*_*K*1_. We found that the critical AR at 1Hz was *AR* ≈ 6.0. However, if we did the same for the repolarization phase only (25% *G*_*K*1_ for *dV*/*dt* < 0 and 100% *G*_*K*1_ for *dV*/*dt* > 0), the effect of the partial block of *I*_*K*1_ completely disappeared, and the block occurred at *AR* ≈ 3.7. Hence, we concluded that the change in the AR occurred because of the effects of *I*_*K*1_ during the upstroke of the action potential. To locate the exact phase of the upstroke, where the effect of *I*_*K*1_ is most important, we blocked it separately either before or after activation of *I*_*Na*_. In our model, *I*_*Na*_ is activated if *V* > −50 *mV*. Therefore, we conducted similar simulations in which we either blocked *I*_*K*1_ on the upstroke when *I*_*Na*_ ≈ 0 (*V* < −50*mV*), or we did it for nonzero *I*_*Na*_ when *V* > −50*mV*. We found that in the first case, when *I*_*K*1_ was blocked before activation of *I*_*Na*_, the critical *AR* ≈ 6.0, while in the second case, when *I*_*K*1_ was blocked after activation of *I*_*Na*_, *AR* ≈ 3.7. Hence, we concluded that in normal conditions *I*_*K*1_ makes it more difficult to reach the excitation threshold of the tissue, and hence the reduction of *I*_*K*1_ makes front propagation possible over a wider range of AR. This can also explain why *I*_*K*1_ current, whose value is small compared to *I*_*Na*_, can still have a substantial effect on wave propagation.

#### Hyperkalemia

**Fig 7:**
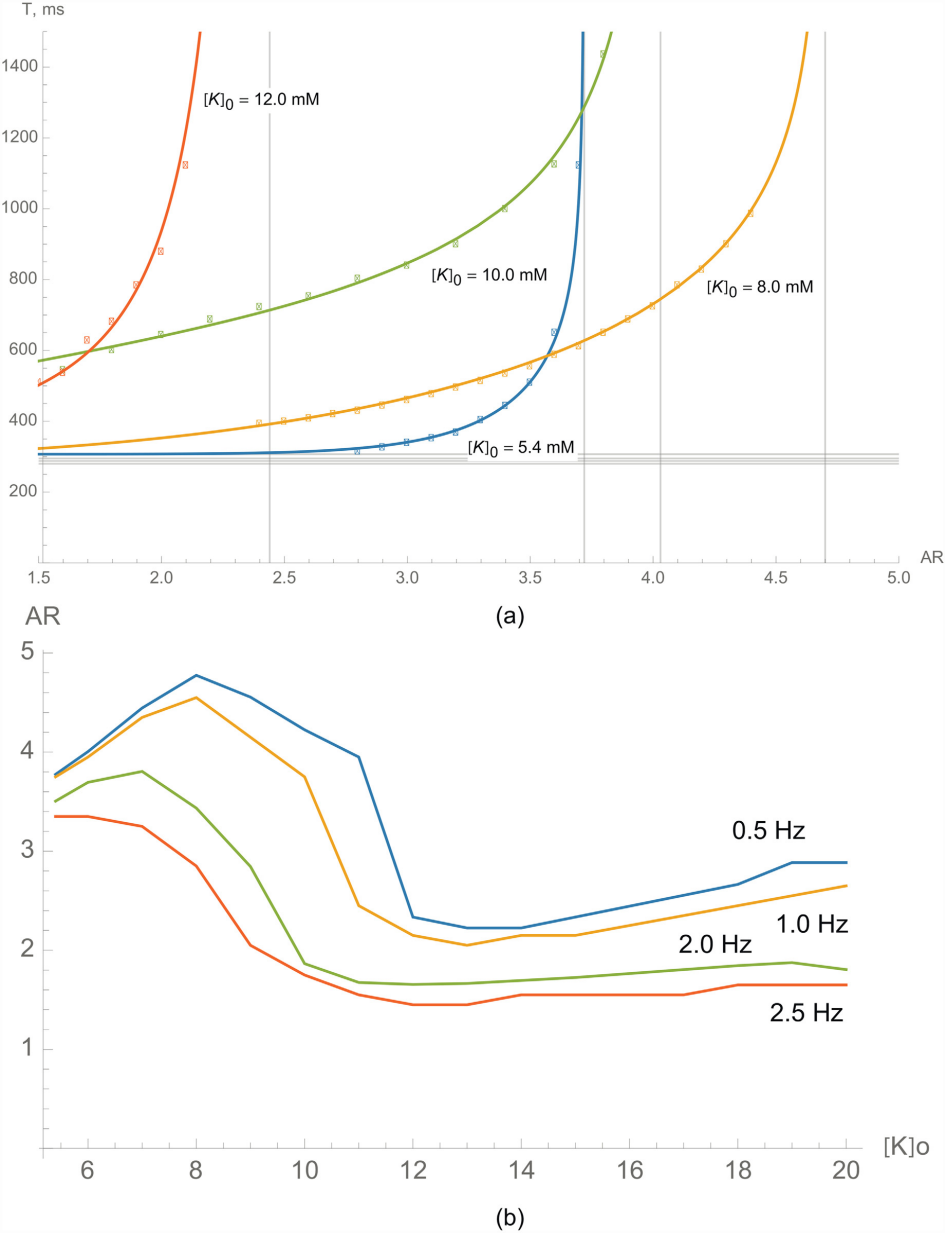
Conditions for waveblock formation in hyperkalemia. a) The dependence of the critical period of stimulation on the AR for various potassium concentrations outside of the cell [*K* ^+^]_*o*_. The blue line shows the critical period of stimulation under normal conditions ([*K* ^+^]_*o*_ = 5.4 mM). b) The dependence of the critical AR on potassium concentration outside of the cell [*K* ^+^]_*o*_ for various stimulation frequencies.

Finally, we studied the effect of hyperkalemia (Fig 7), one of the most important consequences of ischemia. Since most cardiac arrhythmias occur under ischemic conditions, the influence of such conditions on block formation is important. We have found that an increase of the extracellular *K* ^+^ concentration results in a complex, nonmonotonous change in critical anisotropies (Fig 7a). Before we analyze the curves shown in this figure, let us consider a more simple representation, shown in Fig 7b, which illustrates the dependency of the critical AR vs. potassium concentration ([*K* ^+^]_*o*_) for several stimulation frequencies. (0.5 Hz, 1 Hz, 2 Hz and 2.5 Hz).

One can see that at low frequencies (0.5 Hz or 1 Hz), the critical AR starts growing from 3.72 under normal conditions to a maximum value of around 4.8 at [*K* ^+^]_*o*_ = 8 mM for 0.5 Hz and 4.6 for 1 Hz. A further increase of [*K* ^+^]_*o*_ until approximately 12 mM results in a steep decline of the critical AR until approximately 2.3 for 0.5 Hz and 2.2 for 1 Hz, which is even lower than under normal conditions. An additional increase of [*K* ^+^]_*o*_ does not influence block formation significantly. At high frequencies of stimulation (2.5 Hz), the critical AR is 3.4 under normal conditions and monotonically decreases to 1.6 with an increase of [*K* ^+^]_*o*_.

This complex behavior can be explained as follows: the increase of [*K* ^+^]_*o*_ shifts the resting potential to less negative values. That, on the one hand, brings the resting potential closer to the excitation threshold. On the other hand, such depolarization results in partial inactivation of the sodium channels. As a result, we see an increase of the critical AR. However, a further increase in [*K* ^+^]_*o*_ substantially decreases the availability of sodium channels, which in turn decreases the excitability of cardiac cells and results in a decrease of the critical AR. These effects are apparent at low stimulation frequency. At high stimulation frequency, however, the sodium current is already partially suppressed, and thus, the first phase of the AR increase is absent.

Now, we can explain the results presented in Fig 7a. The vertical asymptote shifted right to AR = 4.7 at [*K* ^+^]_*o*_ = 8 mM, whereas the horizontal asymptote did not change significantly. This change corresponds with increased excitability, but no change in APD (Fig 3). With a further increase of [*K* ^+^]_*o*_, the vertical asymptote shifted left to AR = 2.44 for [*K* ^+^]_*o*_ = 12 mM, since there were less sodium channels available. The APD in hyperkalemia shortened slightly (Fig 3), which resulted in a vertical downward shift of the horizontal asymptote from 307 ms under normal conditions to 281 ms at [*K* ^+^]_*o*_ = 12 mM.

All the dependencies above were fitted with the following function:

$$\begin{array}{c} T\left(AR\right)=a+\frac{bAR^{c}}{{\left(AR_{1}-AR\right)}^{d}}, \end{array}$$

where *AR*_1_ is a critical anisotropy for a single travelling pulse, *a* is the refractory period at high frequency (short period), and *b*, *c*, and *d* are free parameters. The values of the parameters for all curves shown in Figs 4–7 are presented in Table 1.

**Table 1:** Critical anisotropy ratio and fitting parameters.

Conditions		*AR*_1_	*a*	*b*	*c*	*d*
Normal		3.72	307	0.0014	9.09	0.333
Na (Fig 4a)	75%	3.61	310	0.013	7.56	0.333
	50%	3.41	304	0.1	6.31	0.333
	25%	2.93	290	0.3	6.15	0.333
Ca (Fig 4b)	75%	3.56	264	0.05	6.34	0.429
	50%	3.35	246	0.06	6.20	0.432
	25%	3.15	203	0.09	6.00	0.463
Kr (Fig 5a)	75%	3.72	312	0.0018	8.89	0.362
	50%	3.73	324	0.0026	8.54	0.410
	25%	3.74	333	0.0066	7.74	0.484
Ks (Fig 5b)	75%	3.72	318	0.0037	8.40	0.347
	50%	3.72	339	0.011	7.56	0.349
	25%	3.72	350	1.0	3.96	0.448
K1 (Fig 6)	75%	3.90	310	0.004	7.94	0.333
	50%	4.32	308	0.07	5.42	0.333
	25%	6.20	207	44.95	1.25	0.333
[*K* ^+^]_*o*_ (Fig 7)	8.0 mM	4.70	295	15.50	2.33	0.300
	10.0 mM	4.03	288	340	0.46	0.403
	12.0 mM	2.44	281	189	0.19	1.360

### 0.5 Propagation block and reentry formation

In previous sections, we have shown that a waveblock can occur at the border between areas with orthogonal fiber orientation. Here, we analyze how this waveblock can result in sustained patterns of excitation.

**Fig 8:**
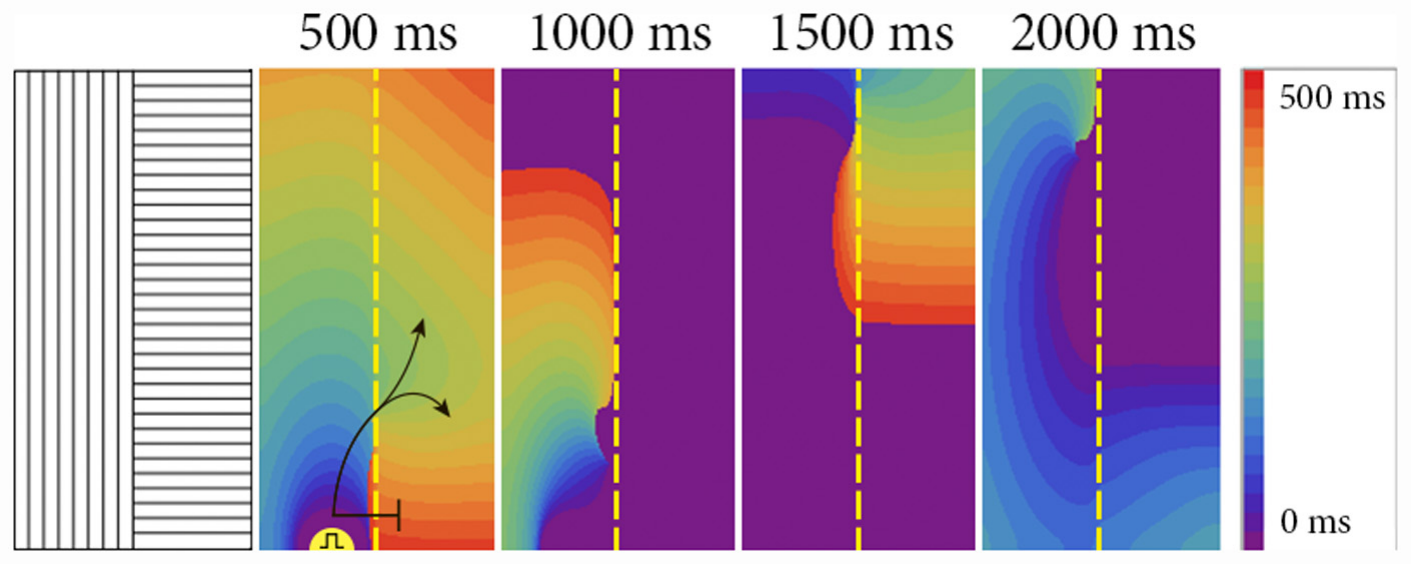
Formation of transient reentry (ectopic beats) at the border between areas with orthogonal fiber orientation. Two stimuli were applied 6 mm from the border with a delay of 500 ms. The AR is 2.0 and [*K* ^+^]*o* is 10 mM. Size of the tissue: 6.4 cm x 3.2 cm.

**Fig 9:**
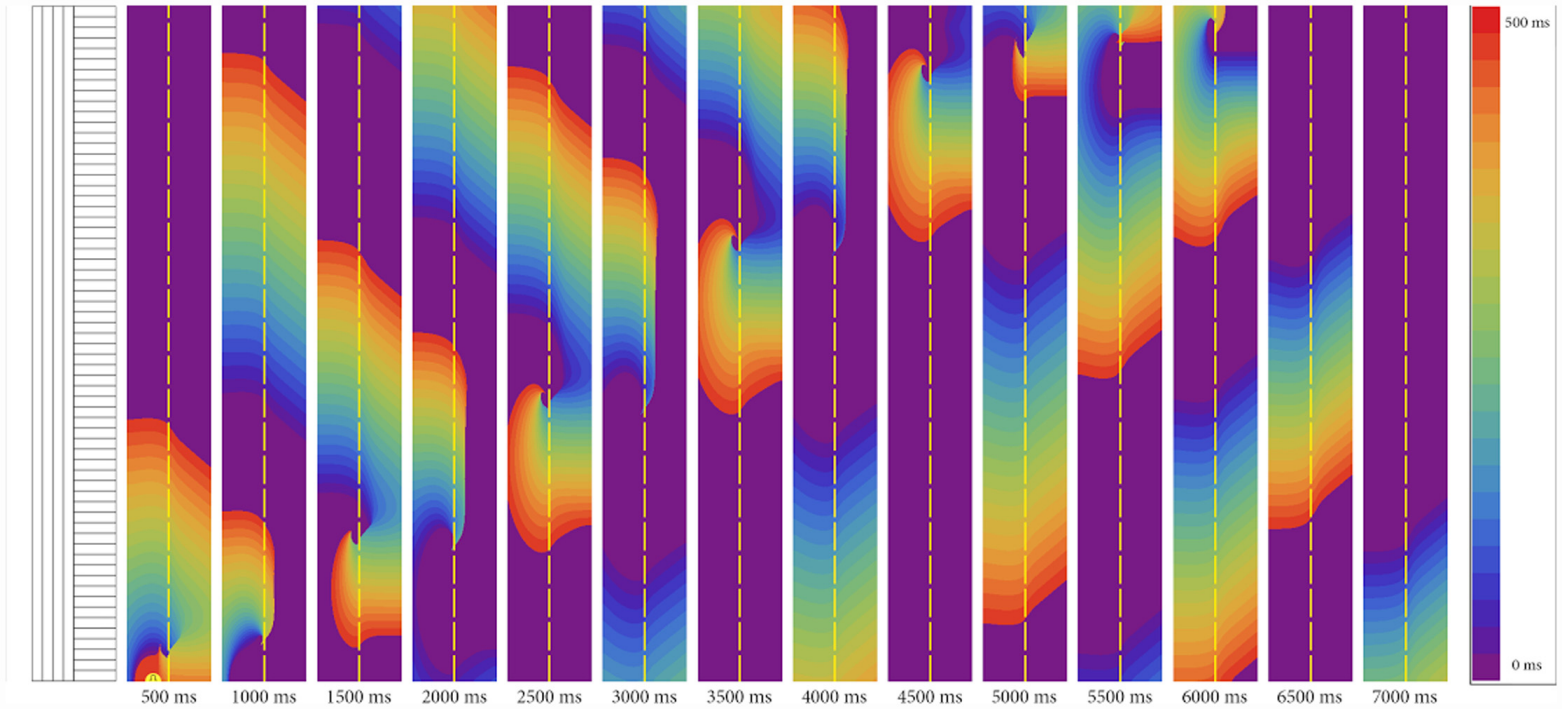
Drift of the reentry. The same simulation as in Fig 8. Size of the tissue: 25.6 cm x 3.2 cm.

**Fig 10:**
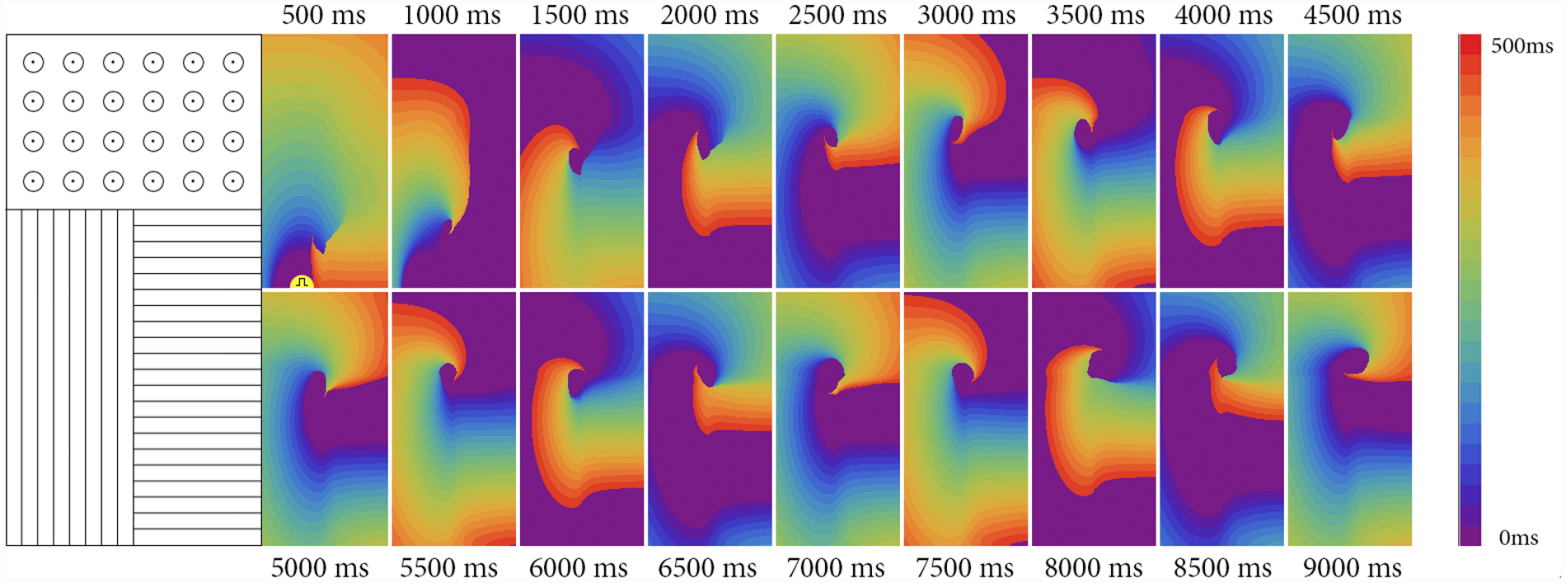
Reentry formation at the border between areas with orthogonal fiber orientation, stabilized at the isotropic area. Two point stimuli were applied 6 mm from the border with a delay of 500 ms. The AR is 2.0 and [*K* ^+^]*o* is 10 mM. Fiber alignment is shown in the left figure. Size of the tissue: 6.4 cm x 3.2 cm.

Fig 8 shows the formation of a transient reentrant source as the result of point stimulation. We see that, initially, the elliptic wavefront reaches the boundary and is blocked there (marked by ⊥ symbol in Fig 8 at 500 ms). However, the distal parts of the front have an incidence angle larger than zero, and thus, changes in the resistance in the incidence direction become smaller. Consequently, it penetrates into the right part of the medium in a location about 1 cm away from the stimulation point (shown by arrows in Fig 8 at 500 ms). Eventually, a reentrant pattern is formed. This pattern, however, is unstable and drifts upwards. To illustrate this drift, we performed the same simulations in a domain that has the same thickness but is 4 times longer vertically. We saw that the drift continued, and the wave finally disappeared at the upper boundary of the tissue (Fig 9). Such a drift is the result of an anisotropy change in the cardiac tissue and can be understood from simple geometrical considerations: faster propagation along the border in the left part than in the right part of the medium results in an overall shift upward for the given direction of rotation (see also [23]). To limit the drift in the next simulation, we added an isotropic area around the border (Fig 10). In this case, reentry, which occurred via similar mechanism, reached the isotropic area and stabilized there. Therefore, we see that a stable reentrant pattern can be formed at the anisotropic boundary due to the waveblock formation mechanism studied in this paper.

All of the above results were obtained in conditions of severe hyperkalemia ([*K* ^+^]_*o*_ = 10 *mM*). It is possible to initiate the reentry in similar systems under normal conditions, but the AR should be two times higher (see Fig 7b for 2 Hz).

## Discussion

In this paper, we performed a detailed study of waveblock formation at the anisotropic boundary. Although this system was already studied using a low dimensional model of a cardiac cell [18], we used an ionic model for human cardiac tissue for the first time to describe the system of abrupt changes in fiber orientation. In addition, we studied in detail how the formation of the waveblock depends on both the period of stimulation and the conductivity of different ionic currents.

Our main conclusion is that such a block is possible, and that it occurs for reasonable values of the AR (between 2.4 and 6.2 with respect to the velocities of propagation). Changes in the conductivity of ionic currents have a substantial effect on the critical AR. From the results explained above, *I*_*K*1_ had the greatest effect on block formation. Suppression of *I*_*Na*_ and *I*_*Ca*_ resulted in 21% and 15% changes in the critical AR, respectively. If we assume that arrhythmias occur via a sink-source mismatch mechanism, then the blockers of the inward rectifier current (*I*_*K*1_) should be the most effective in arrhythmia prevention. On the contrary, class I (blocking *I*_*Na*_) and class IV (blocking *I*_*Ca*_) antiarrhythmic agents increase the probability of block formation, decreasing critical anisotropy and critical frequency. Class III (affecting *I*_*Kr*_) antiarrhythmic drugs play no part in our block formation scenario.

Note that the waveblock in this situation occurs only for the waves propagating from the region with slower propagating velocity to the region with faster propagating velocity, since density of the current decreases at such a boundary. If the wave propagates in the opposite direction (not shown in Fig 2), it can always pass the boundary, because in that case, the density of the current at the boundary increases. In this case, therefore, we have the formation of a so-called “unidirectional block” (i.e., a situation in which the wave is blocked in one direction but can propagate in the opposite direction). The formation of uni-directional blocks is important for the onset of reentrant sources of excitation.

Propagation of the excitation wave across the border between areas with different coupling coefficients was previously studied by Zemlin and Pertsov [24, 25] using the Luo and Rudy model [26] for guinea pig ventricular tissue and the model for canine atrial tissue by Ramirez et al. [27]. In these studies, the transition between the regions with different coupling coefficients was smooth at some border regions and abrupt at others. It was shown that reentry can be formed in such a system due to the partial block of excitation. The scenario was related to the problem of the onset of reentry in pulmonary vein regions during atrial fibrillation and the bradicardiac onset of reentry in the ventricles. Although the setup of the boundary, the anisotropy, and the model used [24, 25] were different, the biophysical waveblock mechanism observed in [24, 25] is similar to that studied in our paper. Therefore, it would be interesting to find out how the changes in ionic currents researched in our study would affect the processes of reentry formation in the setup [24, 25], and what potential effects may occur for atrial fibrillation.

It would be illuminating to study the conditions for waveblock formation described in this paper in an experiment. One could conduct it by using neonatal cell cultures, grown on specific scaffolds, and by providing preferential direction for the elongation of the cells, as we did in our previous work [18]. By varying the AR in the samples, it is possible to compare the probability of block formation under normal conditions and with suppressed *I*_*Na*_ (with TTX) and *I*_*Ca*_ (with nifedipine), or in hyperkalemia (in a media with higher *K* ^+^ concentration). For human cardiac cells, a similar study could potentially be done using IPS cells [28].

In this paper, we have studied one of the effects of acute ischemia: hyperkalemia. Another effect of ischemia, which has a pronounced influence on the dynamics of reentrant patterns, is hypoxia [29, 30]. In hypoxia, the duration of APD can be substantially shorter due to the opening of *I*_*K*_*ATP*__, and, consequently, the wavelength of the reentry may be substantially reduced. As a result, the waveblocks formed at the boundary become stable and could also be stabilized in media of smaller size.

We studied the effects of the blocks in a 2D model of cardiac tissue, but in many parts of the heart (e.g., the ventricles), cardiac propagation was essentially in 3D. Thus, it is helpful to understand the mechanisms of waveblock formation in 3D as well. From a general theoretical point of view, plane wave propagation in 3D is quasi 2D. In that situation, therefore, simulations performed in our study can be applied for some 3D effects. For curved 3D wavefronts, theory indicates that the curvature effects depend on the mean curvature of the wavefront [31, 32], meaning that the curvature effect on the wave propagation in 3D will be even more essential than in 2D. Thus, waveblock formation in similar conditions is more feasible in 3D.

In this paper, we have studied block formation at the boundary of an abrupt change of fibers in homogenous cardiac tissue. However, in addition to anisotropy, other factors are also important for block formation. One of these factors is the heterogeneity of cardiac tissue ([33]). Changes in the conductivity of various ionic channels, in addition to the effects of block formation on the border between anisotropic areas, may also result in a change of the heterogeneity of cardiac tissue. For example, Colman et al. [34] showed that channel blockers for *I*_*K*1_ and *I*_*Kr*_ increase electrical heterogeneity and play a pro-arrhythmogenic role, in spite of the fact that they also increase the wavelength and therefore display an anti-arrhythmoginic effect. Increased heterogeneity also results in reentry instability [34, 35]. In light of that fact, it would be interesting to study the combined effect of reduced ionic currents on the onset of arrhythmias in a setup that combines anisotropy changes with APD heterogeneity.

### Limitations

We did not use a bidomain description for the cardiac tissue. A recent study [36] showed that for normal propagation and recovery, the monodomain and bidomian approaches give similar results. We expect this to remain true for our studies on propagation block. However, this statement needs to be validated in subsequent studies. We only studied the effects for a simple geometry of the boundary: an anisotropy change along a straight line. It would be enlightening to also study similar effects for a more complex boundary with a curvature and find out if the more complex boundary has any effect on the block formation conditions. We performed our simulations using a TP06 model of cardiac tissue. It would be interesting to study similar phenomena for other models of human and animal cardiac cells, such as in [37–39]. We also performed simulations with 2D models of homogeneous cardiac tissue. It would be beneficial to conduct similar studies in 3D, with anatomically accurate geometries, and in the presence of ionic heterogeneities of cardiac tissue.

## Supporting Information

S1 Table APD restitution data for various conductivities of ionic currents and external [*K* ^+^]_*o*_ concentration. (List 1) Inhibition of *I*_*Na*_; (List 2) Inhibition of *I*_*Ca*_; (List 3) Inhibition of *I*_*Ks*_; (List 4) Inhibition of *I*_*Kr*_; (List 5) Inhibition of *I*_*K*1_; (List 6) Hyperkalemia. The table contains the dependency of *APD*_90_ on the period of stimulation. Frequencies higher than 2.7 Hz were obtained by gradually decreasing the period by 5 ms per cycle. These data were used in Fig 3.
(XLSX)

S2 Table Critical AR for various conductivities of ionic currents and external [*K* ^+^]_*o*_ concentration. The table contains pairs of periods of stimulation (*T*_*pass*_ and *T*_*block*_), that constrain the critical period of stimulation. Wave propagation through the border is possible for all stimulation periods *T* ≥ *T*_*pass*_, and block occurs for every *T* ≤ *T*_*block*_. (List 1) contains dependencies of critical periods (*T*_*pass*_, *T*_*block*_) on AR with fixed conductivity or external [*K* ^+^]_*o*_ concentration; (List 2) contains dependencies of AR on [*K* ^+^]_*o*_ with fixed stimulation frequencies. These data were used in Figs 4–7.
(XLSX)
